# Duodenal enteroglucagonoma revealed by differential comparison of serum and tissue glucagon reactivity with Siemens' *Double Glucagon Antibody *and DakoCytomation's *Polyclonal Rabbit Anti-Human Glucagon*: a case report

**DOI:** 10.1186/1752-1947-4-178

**Published:** 2010-06-15

**Authors:** Wesley B Vanderlan, Ziying Zhang, Marwan S Abouljoud

**Affiliations:** 1 Department of Surgery, Division of Transplant Surgery and Hepatobiliary Surgery, Henry Ford Hospital, West Grand Boulevard (CFP-2), Detroit, MI 48202, USA; 2 Department of Pathology, Henry Ford Hospital, West Grand Boulevard, Detroit, MI 48202, USA

## Abstract

**Introduction:**

This case report demonstrates that the differential immunohistochemical reactivities of Siemens' *Double Antibody Glucagon *compared to DakoCytomation's *Polyclonal Rabbit Anti-Human Glucagon *allow for pathologic distinction of enteral versus pancreatic glucagonoma.

**Case presentation:**

A 64-year-old Caucasian man was diagnosed with a duodenal enteroglucagonoma following presentation with obstructive jaundice. He had a low serum glucagon level using Siemens' *Double Antibody Glucagon*, a clinical syndrome consistent with glucagon hypersecretion. A periampullary mass biopsy proved to be a neuroendocrine tumor, with positive immunohistochemical reactivity to DakoCytomation's *Polyclonal Rabbit Anti-Human Glucagon*.

**Conclusions:**

Differential comparison of the immunohistochemical reactivities of Siemens' *Double Antibody Glucagon *and DakoCytomation's *Polyclonal Rabbit Anti-Human Glucagon *discerns enteroglucagon from pancreatic glucagon.

## Introduction

Pancreatic glucagonomas are rare neuroendocrine tumors with an estimated incidence of approximately 1 in 20 million [1]. Duodenal glucagonomas are reported, but are even rarer with an unknown true incidence [2]. Necrotizing migratory erythema (NME), glucose intolerance, weight loss and anemia form a presenting constellation known as the glucagonoma syndrome. Patients most commonly present in the sixth decade of life with an even gender distribution. Tumors usually present larger than 4 cm and are commonly associated with metastasis in over 50% of patients [3,4].

Neuroendocrine tumors are slow-growing neoplasms [1]. Both chronic and acute pancreatitis has been associated with neuroendocrine tumors [5]. NME presents on average 7 years before the diagnosis of glucagonoma [4,6]. Diabetes mellitus is commonly diagnosed 5 years before the ultimate diagnosis of glucagonoma [6].

Treatment of glucagonomas depends on location, size and lymph node involvement. Simple excision with resection of peripancreatic lymph nodes is the treatment of choice for isolated confined lesions [7]. Periampullary, large and metastatic tumors require more extensive resection [7]. Surgical debulking has demonstrated decreased morbidity and increased survival [3]. For advanced lesions, octreotide is the treatment of choice for symptomatic control [7].

"Enteroglucagon" applies to a group of proglucagon peptide cleavage fragments. The proglucagon moiety forms the precursor molecule for both pancreatic and enteral glucagon. Differential proteolytic processing occurs in these tissue spaces. Enteral cleavage results in the major products glucagon-like peptide 1 (GLP-1), glucagon-like peptide 2 (GLP-2) and glicentin. Glicentin is cleaved to oxyntomodulin and glicentin-related pancreatic product (GRPP). Oxyntomodulin closely resembles glucagon with the only difference being an eight amino acid C-terminus extension [8]. The glucagon receptor has only a 2% affinity for oxyntomodulin but does exhibit some limited glucagon-like bioactivity [8]. GRPP is formed in both pancreatic and intestinal tissues.

Enteroglucagon secreting tumors have been reported but detection of enteroglucagon was indirect [2,9,10]. Stevens *et al*. specifically reported assay positivity for an N-terminal reactive antibody measuring both pancreatic and gut glucagon-like immunoreactivity with negative C-terminal reactive antibody more specific for pancreatic glucagon and demonstrating only a 2% cross-reactivity with gut extracts [9]. These tumors were clinically diagnosed and treated as glucagonomas [2,9,10].

### Case presentation

Our patient was a 64-year-old Caucasian man referred from an outside hospital for evaluation and treatment of obstructive jaundice secondary to a periampullary mass. Patient informed consent for publication of findings was obtained during admission. Prior admission to the outlying hospital had occurred 5 days before transfer following presentation with jaundice, fatigue and bilateral lower extremity edema.

Hypoalbuminemia, refractory severe hypokalemia, uncontrolled new onset diabetes mellitus, and anemia were associated findings. New onset diabetes mellitus was diagnosed 3 weeks before developing jaundice. His medical history was significant for gout, hypertension, hyperlipidemia, osteoarthritis, degenerative disk disease, erectile dysfunction, and two episodes of idiopathic pancreatitis 15 years earlier. Glycosylated hemoglobin measured an elevated 7.5%. Endoscopic retrograde cholangiopancreatography (ERCP) was performed at the referring institution before transfer. Biopsies returned a diagnosis of neuroendocrine tumor, not otherwise specified. The patient was then transferred to our hospital. His serum potassium remained low despite all replacement efforts. Hypoalbuminemia worsened to 1.6 gm/dL. Serum glucagon was measured using Siemens' *Double Glucagon Antibody *and found to be depressed (37 ng/L; normal 40-130 ng/L). The serum serotonin level (< 25 ng/mL) was low (normal 90-195 ng/L) and the vasoactive intestinal polypeptide (VIP) level (< 30 pg/mL) was within normal limits (normal < 100 ng/L). Carcinoembryonic antigen (CEA) level was normal at 1.1 ng/mL. Carbohydrate antigen 19-9 (CA 19-9) was markedly elevated at 233.5 U/mL. Computed tomography revealed a 3.2 cm by 1.9 cm hypervascular mass in the medial duodenal wall juxtaposing the pancreas. Three hepatic lesions appeared consistent with metastasis.

Exploratory laparotomy was performed. Five perihepatic lymph nodes appeared suspicious for metastatic disease; they were surgically excised and examined by frozen section. Three of these five lymph nodes were positive for metastatic neuroendocrine carcinoma. Two liver masses each less than 1 cm were identified by intra-operative ultrasound. Each lesion was wedge resected and pathologic examination confirmed metastases. Pancreaticoduodenectomy was indicated and performed due to lymph node and hepatic metastases. Pathology specimens stained positive for chromogranin, synaptophysin, and glucagon. The tumor mass was 4.5 cm and infiltrated through the duodenal wall into the pancreas. Extensive angiolymphatic invasion was noted.

The patient's post-operative course was complicated by respiratory aspiration, disseminated candidiasis with candidal sepsis, wound infection, disseminated varicella, atrial fibrillation, acute renal failure and a subhepatic intra-abdominal candidal abscess. Discharge to a rehabilitation center occurred on post-operative day 53. Results of the multidisciplinary gastrointestinal tumor board recommended close monitoring with long-acting octreotide.

## Discussion

The patient presented clinically with evidence supporting a glucagonoma. The classic glucagonoma syndrome consists of necrotizing migratory erythema, hyperglycemia without ketosis, weight loss, and anemia [1,4,11]. Uncontrolled new onset diabetes mellitus without ketosis, normochromic and normocytic anemia, and hypoalbuminemia were recognized in this patient on admission. Peripheral edema was present in our patient and has been reported in association with glucagonomas and enteroglucagonomas [9,10]. Tissue biopsy confirmed a neuroendocrine tumor origin and the absence of an associated tumor within the pancreas solidified a duodenal origin.

The Whipple resection specimen consisted of a segment of distal stomach, duodenum, and proximal pancreas. Located in the immediate periampullary region was a 4.5 × 2.0 × 1.5 cm well-circumscribed solid firm tan-yellow submucosal nodule covered by intact mucosa. Light microscopy demonstrated the tumor infiltrating through the duodenal wall into the superficial pancreas. On immunohistochemistry, tumor cells were positive for synaptophysin, chromogranin and glucagon. The manufacturer reported that the *Polyclonal Rabbit Anti-Human Glucagon *antibody reacts with all moieties containing the glucagon molecule *(DakoCytomation, Inc.: Package Insert and Laboratory Notes, Immunogen: Human Glucagon (Polyclonal Rabbit Anti-Human Glucagon). Carpinteria, California: DakoCytomation, Inc.) *We sought to clarify the inconsistencies in the serum and tissue glucagon reactivities.

The analyzing laboratory was contacted and no compromise of the patient's submitted specimen was suspected. Prior studies performed by Siemens (Los Angeles, CA, USA) showed that the *Double Antibody Glucagon *I^125 ^radioimmunoassay used for serum glucagon level determination failed to detect any glucagon reactivity with the addition of Glucagon-like Peptide 1 (GLP-1), Glucagon-like Peptide 2 (GLP-2), or Oxyntomodulin (Glucagon 37/Enteroglucagon) to a level of 1,000,000 pg/ml *(Siemens, Inc.: Package Insert, Double Antibody Glucagon. Siemens Los Angeles, California)*. Sensitivity for human pancreatic glucagon, however, was excellent. The Siemens' *Double Antibody Glucagon *I ^125 ^radioimmunoassay then has particular specificity for pancreatic glucagon but not enteral glucagon. Consequently, analysis of the patient's serum only indicated the level of pancreatic glucagon while tissue reactivity with *Polyclonal Rabbit Anti-Human Glucagon *reagent was capable of detecting both pancreatic and enteral glucagon. To summarize, the laboratory blood and solid tissue results revealed an intestinal neuroendocrine tumor producing a non-pancreatic form of glucagon: enteroglucagon. GLP-1 inhibition of glucagon secretion may have resulted in the depressed serum level of detectable pancreatic glucagon [8].

## Conclusion

Serum glucagon levels measured with *Double Antibody Glucagon *reflect only pancreatic glucagon production and do not indicate enteroglucagon production. Tissue analysis using *Polyclonal Rabbit Anti-Human Glucagon *detects any glucagon sequence containing moieties including enteroglucagon. Patients clinically exhibiting a glucagonoma syndrome with paradoxical low serum glucagon levels and tissue glucagon immunohistochemical positivity may possess an underlying enteroglucagonoma.

## Consent

Written informed consent was obtained from the patient for publication of this case report. A copy of the written consent is available for review by the Editor-in-Chief of this journal.

## Competing interests

The authors declare that they have no competing interests.

## Authors' contributions

WV directly assembled the patient information, formulated in depth analyses of the known literature, performed the operative interventions and managed the recovery of the patient, constructed and edited the manuscript, was responsible for the final construction of the manuscript for submission to the senior staff/primary investigator, and served as the corresponding author. ZZ processed, evaluated and diagnosed the submitted surgical specimens. She also constructed the pathology narrative for the final manuscript. MA served as the senior staff reviewer/primary investigator. He directly supervised every element of the patient's course and was responsible for final approval of the submitted manuscript following his directed revisions.
